# Magnetic Resonance-Guided Laser Interstitial Thermal Therapy (MR-gLiTT) in Pediatric Epilepsy Surgery: State of the Art and Presentation of Giannina Gaslini Children's Hospital (Genoa, Italy) Series

**DOI:** 10.3389/fneur.2021.739034

**Published:** 2021-10-26

**Authors:** Alessandro Consales, Erica Cognolato, Mattia Pacetti, Maria Margherita Mancardi, Domenico Tortora, Giuseppe Di Perna, Gianluca Piatelli, Lino Nobili

**Affiliations:** ^1^Unit of Neurosurgery, Istituto di Ricovero e Cura a Carattere Scientifico (IRCCS) Istituto Giannina Gaslini, Genoa, Italy; ^2^DINOGMI, University of Genoa, Genoa, Italy; ^3^Unit of Child Neuropsychiatry, Istituto di Ricovero e Cura a Carattere Scientifico (IRCCS) Istituto Giannina Gaslini, Genoa, Italy; ^4^Unit of Neuroradiology, Istituto di Ricovero e Cura a Carattere Scientifico (IRCCS) Istituto Giannina Gaslini, Genoa, Italy; ^5^Unit of Neurosurgery, Azienda Ospedaliera Universitaria (AOU) Città della Scienza e della Salute, University of Turin, Turin, Italy

**Keywords:** epilepsy surgery, pediatric, laser, magnetic resonance, interstitial, MR-gLiTT

## Abstract

Magnetic resonance-guided laser interstitial thermal therapy (MR-gLiTT) is a novel minimally invasive treatment approach for drug-resistant focal epilepsy and brain tumors. Using thermal ablation induced by a laser diode implanted intracranially in a stereotactic manner, the technique is highly effective and safe, reducing the risk associated with more traditional open surgical approaches that could lead to increased neurological morbidity. Indications for MR-gLiTT in pediatric epilepsy surgery include hypothalamic hamartoma, tuberous sclerosis complex, cavernoma-related epilepsy, SEEG-guided seizure onset zone ablation, corpus callosotomy, periventricular nodular heterotopia, mesial temporal lobe epilepsy, and insular epilepsy. We review the available literature on the topic and present our series of patients with drug-resistant epilepsy treated by MR-gLiTT. Our experience, represented by six cases of hypothalamic hamartomas, one case of tuberous sclerosis, and one case of dysembryoplastic neuroepithelial tumor, helps to confirm that MR-gLiTT is a highly safe and effective procedure for several epilepsy conditions in children.

## Introduction

Magnetic resonance-guided laser interstitial thermal therapy (MR-gLiTT) is a novel and very promising minimally invasive therapeutic approach in the field of neurosurgery. The two major indications for this type of neurosurgical treatment are drug-resistant focal epilepsy and brain tumors (1). LiTT uses heat to generate ablation by light absorption of tissue (2). The physical principle of LiTT is based on the denaturation of tissue proteins, which starts if a temperature above 50°C is applied for a few seconds (3). The use of lasers is not entirely new in neurosurgery. The real novelty of LiTT is that it is a stereotactic method that involves the use of a laser diode implanted intracranially, so it does not require a craniotomy but simply a very small hole in the skull. Another distinguishing feature, compared with techniques that employ the same physical principle as LiTT (e.g., radiofrequency thermocoagulation), is that LiTT is not only spatially driven by MR but it is continuously controlled by it, due to the fact that all components of the LiTT equipment are MR-compatible (4). Each type of tissue has an energy absorption coefficient, called proton resonance frequency (PRF) (5). Pathological brain tissue possesses a higher PRF. This promotes ablation of abnormal tissue, the target of treatment, while normal tissue is spared. This allows the formation of a transition zone of approximately 1 mm between the ablation zone and normal brain tissue, which is monitored in near real time by MR thermometry (6, 7). MR-gLiTT uses synchronized T1-weighted images. Once the laser is activated, the hydrogen bonds decrease in the area of ablation. This determines an increase in tissue penetrability. MR thermography measures temperature differences by subtracting thermal fast-spoiled gradient-recalled phase images obtained after administration of thermal energy from a reference fast-spoiled gradient-recalled phase image obtained at body temperature before any energy pulse is delivered; therefore, accuracy on baseline temperature is critical to the software's ability to predict the ablation damage (8). The thermal mapping is updated every 3, 6, and 8 s for single, biplanar, and triplanar viewing, respectively (9).

There are two LiTT systems available on the market: the Visualase system (Medtronic, Minneapolis, MN, USA) and the NeuroBlate system (Monteris Medical Inc., Plymouth, MN, USA), the latter only marketed in North America so far. Both systems use color-coded thermal maps superimposed on MR and have a software that, using the Arrhenius equation that estimates cell death based on the temperature and time dependence of protein denaturation processes, represents areas of irreversible damage (7). The estimated zone of tissue necrosis is shown in near real time as an orange zone with the Visualase system and as yellow, blue, and white lines with the NeuroBlate system.

MRgLiTT has been licensed for use in Europe in spring 2018.

In this paper, we report on the state of the art on the use of MRgLiTT in pediatric epilepsy surgery. Moreover, we present the case series of patients with focal epilepsy associated with lesions treated by MRgLiTT at Giannina Gaslini Children's Hospital in Genoa (Italy).

## Principal Indications In Pediatric Epilepsy Surgery

### Hypothalamic Hamartoma (HH)

Hypothalamic hamartomas (HHs) are rare, tumor-like, and non-progressive malformative lesions that occur during fetal development from ventral hypothalamus (10). Although there is great interindividual variability in the clinical picture, symptoms generally occur during childhood or adolescence and are mainly characterized by endocrinological disorders (especially precocious puberty), epilepsy, and cognitive–behavioral disorders, according to their anatomical location (11). Epilepsy usually occurs during the first year of life and is characterized by gelastic or, less frequently, dacrystic seizures. Several classifications have been proposed for HHs with the aim to guide the best surgical approach and to predict seizure and functional outcome after surgery; among these, the classification of Delalande remains the most widely adopted at present (12). These classifications play a less relevant role in MR-gLiTT, which can also be planned in multiple steps for particularly voluminous lesions (13). Treatment of HH-associated epilepsy using LiTT is providing extremely encouraging results, both when evaluated in absolute terms and in comparison with other treatment options (e.g., open surgery, endoscopic, radiosurgery) (1, 14). In a recent review, seizure control achieved by LiTT in patients with a follow-up of at least 1 year was 87% in patients with gelastic seizures and 60% in patients with other type of seizures (15). These data, in themselves very good, are even more relevant when compared with the other forms of treatment mentioned above, taken as a whole. In these forms, in fact, with follow-up of comparable duration, the share of patients who are seizure-free is about one-third (14, 16). LiTT can be used both as a first treatment of HH and in cases already treated unsuccessfully with other methods. In this regard, it is important to note, for LiTT as for other treatment options, that disconnection of the hamartoma from the epileptogenic network, rather than its ablation or resection, is often sufficient to achieve seizure freedom (17).

Complications with LiTT are often transient but can be severe. In their recent series of 18 patients, Xu et al. (18) reported a 39% (7/18) incidence of new neurological deficits (including hemiparesis and visual disturbances) and an 11% (2/18) incidence of short-term memory problems as immediate complications. At the last follow-up, many patients with initial neurologic deficits had improved, with 22% (4/18) having persistent deficits but only 1 (6%) having functional impact. Hypothyroidism was the only long-term endocrine deficit (11%, 2/18). Over time, some patients (22%, 4/18) reported new subjective problems with short-term memory, weight gain, or increased appetite. Memory problems can be caused by a mono- or bilateral involvement of the mammillothalamic tract which, as it is known anatomically, is part of the Papez circuit connecting the mammillary body to the anterior thalamic nucleus, although some authors believe that mnestic dysfunctions occur more frequently in patients who have already undergone other types of epilepsy surgery (19). Other adverse events include intracranial hemorrhage and electrolyte imbalance (15). However, since LiTT is a technology that has yet to be widely deployed in the neurosurgical field, a learning curve factor must also be considered. On the other hand, the same complications described for LiTT can occur, even when the known therapeutic alternatives are used, at rates >30% (17).

### Tuberous Sclerosis Complex (TSC)

TSC is a neurocutaneous syndrome that variably involves the brain, skin, kidney, heart, and lungs. Epilepsy is the most common clinical manifestation of TSC, occurring in approximately 90% of cases (20). Epileptogenesis has been theorized to result from different morphological and molecular abnormalities observed in the cortical tubers and the perituberal cortex (21). The cortical tubers are often multifocal and located within deep brain structures. The anatomical features of these lesions make LiTT a valid therapeutic option because, through this technique, it is possible to treat multiple epileptogenic lesions without the need to perform multiple craniotomies. Tovar-Spinoza and colleagues reported on seven patients with TS and drug-resistant epilepsy who underwent LiTT of cortical tubers. Two patients had a single procedure, and five patients had staged procedures. All of the patients had a meaningful reduction in seizure frequency, and more than 70% experienced a reduction in antiepileptic medications. Three of the four patients who presented with neuropsychiatric symptoms had some improvement in these domains after laser ablation, although the authors did not have data from formal neuropsychological evaluation to support their observations. No perioperative complications were noted. The authors stated that laser ablation represents a minimally invasive alternative to resective epilepsy surgery and is an effective treatment for refractory epilepsy due to cortical tubers (22).

### Cavernoma-Related Epilepsy

Cavernomas are mulberry-like vascular malformations often found in brain and spinal cord. Brain cavernomas can determine irritation (epilepsy) or deficiency symptoms (23). LiTT has increasingly been offered as an alternative minimally invasive treatment for cavernoma-related epilepsy (24, 25). The published case histories are currently quite small in number. However, the very satisfactory epileptological outcome reported in the aforementioned case reports, coupled with excellent overall clinical conditions [e.g., Engel class I in 80% of patients and zero adverse events reported by McCraken et al. (25)], makes it important to continue studying the use of LiTT in the treatment of cavernoma-related epilepsy.

### SEEG-Guided Seizure Onset Zone Ablation

The SEEG is a method of Functional and Stereotactic Neurosurgery that allows an invasive EEG study in order to identify the seizure onset zone (SOZ) in cases where non-invasive diagnostic studies have not produced good anatomo-electro-clinical correlations (26). More recently, it has also been used as a possible therapeutic weapon, for example in radiofrequency thermocoagulation of heterotopic cortical nodules (27). A topic closely related to SEEG studies is that of MR-negative epilepsies, which are a crucial field of investigation in Epilepsy Surgery. When, in MR-negative epilepsies a SOZ is identified and delineated by SEEG, ablation of the SOZ by LiTT can be considered in selected cases (1). Of course, further studies will be needed to define the potential role of a SEEG-guided LiTT in the thermoablative treatment of a SOZ.

### Corpus Callosotomy

Huang et al. recently published a retrospective study of a case series of six patients (three children and three adults) who underwent callosotomy using LiTT. Engel outcomes for completion corpus callosotomy by LiTT were similar to reported outcomes of open completion callosotomy, with seizure reduction primarily observed in adult patients (28). As with other new indications for LiTT, future in-depth studies will be needed for callosotomy using LiTT.

### Periventricular Nodular Heterotopia

Periventricular nodular heterotopias (PNHs) are malformations of cortical development characterized by disorganized but histologically normal aggregates of neuronal and glial cells. They are often associated with drug-resistant epilepsy (29). The anatomic electro-clinical characteristics of PNHs have prompted consideration in the relevant literature of a number of minimally invasive approaches, such as stereotactic radiosurgery and stereotaxy-guided radiofrequency lesioning. Moreover, the possibility of treating these malformations, after adequate epileptological diagnostic procedure, by a minimally invasive surgical approach such as LiTT, especially when they are located in high functional areas, has already been described (30). While data concerning seizure outcome within the pediatric population are still somewhat limited, results in the adult population are very good, with seizure freedom up to 100% and no adverse events after LiTT treatment (1). These data are very striking, as epilepsy involving a PNH can be multifocal, with complex and distributed epileptogenic networks. However, focal resections/ablations can be successful if the role of the PNH within the epileptogenic network is understood. It is therefore more than reasonable to assume that as Epilepsy Surgery centers will increase their experience with LiTT, the trend of LiTT treatment outcomes of PNHs will be the same or even more positive within the pediatric population.

### Mesial Temporal Lobe Epilepsy

Treatment of mesial temporal lobe epilepsy (MTLE) by means of LiTT has been addressed by some studies concerning the adult population (31, 32). It can be reasonably assumed that this was for epidemiological reasons related to this type of epilepsy. Nevertheless, the technical considerations can be considered applicable to the pediatric population as well. In MTLE, the percentage of patients seizure-free after LiTT is lower than in cases treated with open surgery (about 50%) (8). It can be speculated that this is partly due to the particular complexity of the epileptogenic network in MTLE, which may consequently lead to partial or complete error in identifying the target of LiTT. However, it is important to note that LiTT treatment can be repeated, even multiple times, and that it does not preclude further treatment, surgical or otherwise (33).

### Insular Epilepsy

Insular epilepsy is another potentially interesting area of application of LiTT in pediatric epilepsy surgery (34). The insula is indeed a deep encephalic structure with a rich and complex vasculature (35). Therefore, an open surgical approach to the insula, in addition to requiring considerable technical expertise, may be burdened by significant ischemic complications. Perry et al. (36) described 20 pediatric patients with insular epilepsy who underwent 24 LiTT procedures. After a mean follow-up of 20.4 months after their last treatment, 10 patients (50%) were in Engel Class I, 1 (5%) in Engel Class II, 5 (25%) in Engel Class III, and 4 (20%) in Engel Class IV at the last follow-up. Patients were discharged within 24 h of the procedure in more than 60% of cases. Transient complications were registered after seven (29%) procedures: mild hemiparesis in six cases (all patients experienced complete resolution or had minimal residual dysfunction by 6 months), and expressive language dysfunction in another one (resolved by 3 months). More recently, another recent study by Hale et al. (37) compared LiTT and surgical resection of at least some portion of the insular cortex, concluding that both surgical resection and LiTT are valid management options in the treatment of medically refractory insular/opercular epilepsy in children. At present, therefore, and pending further and more in-depth studies, LiTT can certainly be considered an effective and low-risk alternative to open surgery for insular epilepsy.

### Other Epileptogenic Lesions

Focal cortical dysplasias and dysembryoplastic neuroepithelial tumors (DNETs) are the most frequent causes of drug-resistant epilepsy in children (38). Nevertheless, the literature data on the use of LiTT in the treatment of these diseases are still scarce and patchy, when compared with other epileptogenic conditions, such as HH (30, 39–41). Other conditions associated with drug-resistant epilepsy, the treatment of which by LiTT has been sporadically reported to date, are Rasmussen's encephalitis and parasitic lesion (30, 39).

## MR-gLiTT in Pediatric Epilepsy Surgery: Outlines of Technical Principles

The use of MR-gLiTT in Epilepsy Surgery requires the placement, through a micro-hole drill, of a laser fiber within an intracranial lesion target using stereotactic methods (frameless, robotic, or frame-based). A skull anchoring system is mounted on a steel rod and is screwed into the microhole at the calculated angle, forming a solid anchor point for insertion of the laser fiber. The laser probe is then positioned through the above system until it reaches the intracranial target. Heat delivery is monitored in near real time by MR thermography, as explained above, until the desired ablation is achieved; multiple heat deliveries may be required during the single procedure, and repositioning of the laser probe may be necessary (2).

## Giannina Gaslini Children's Hospital Series

Patient demographics, preoperative seizure frequency, seizure semiology, and postoperative outcomes are listed in Table 1.

**Table 1:** Clinical features and outcome of Giannina Gaslini Children's Hospital series.

**Pt**	**Sex**	**Lesion type**	**Age epilepsy onset**	**Epilepsy duration (m)**	**Age at surgery**	**Seizure frequency**	**Semiology**	**EEG pre LITT**	**ASM tried *(n)***	**Co-morbidity**	**Lesion size (mm)**	**Localization**	**Side**	**Laser probe placement system**	**APOS**	**Compl**.	**Last F.U. (months from procedure)**	**Seizure outcome** **(engel** **class)**
#1	M	HH	8 m	99	8 y 11 m	2–3/w	Gelastic seizure, focal w/ impaired awareness seizure	**Interictal**: right CT spikes	1	ID, PP	20 × 18 × 14	Tuber, E.V.	M	Frameless, Medtronic Vertek^TM^	No	No	11	I b
								**Ictal**: bilateral rhythmic SpWs										
#2	M	HH	6 m	15	1 y 9 m	30–40/d	Gelastic seizure, spasms	**Interictal**: bilateral CT SlWs	3	DD, rest tremor	12 × 13 × 13	Tuber/mammillary body, I.V.	L	Frameless, Medtronic Vertek^TM^	No	No	11	Ib
								**Ictal**: uninformative										
#3	M	HH	7 m	20	2 y 3 m	3–4/d	Gelastic and dacrystic seizures	**Interictal**: bilateral SlWs (left>right)	1	Language Delay	18 × 11 × 18	Tuber, I.V/E.V.	L	Medtronic Stealth Autoguide^TM^	No	No	6	Ia
								**Ictal:** no seizures recorded										
# 4	M	HH	36 m	48	7 y	0–10/d	Gelastic, focal with impaired awareness	**Interictal**: right FT SlWs and SpWs	2	Language delay, ID	12 × 10	Tuber, I.V.	R	Medtronic Stealth Autoguide^TM^	No	No	2	I b
								**Ictal**: right FT SpWs										
#5	M	TS (2)	10 m	60	6 y 8 m	1–10/d	Focal with preserved awareness	**Interictal**: right T SlWs	6	ASD	8 × 6, 13 × 10	Temporal lobe neocortex	R	Frameless, Medtronic Vertek^TM^	Yes	No	2	II b
								**Ictal**: Right T EEG flattening followed by lSWs										
#6	F	DNET	60 m	120	15 y 5 m	1/m	Focal with preserved awareness	**Interictal**: Left, CP sharp waves	2	Cardiac malf., hypothyroidism	13 × 10,5 × 14	Parietal	L	Medtronic Stealth Autoguide^TM^	Yes	No	1	Ia
#7	F	HH	0 d	12	11 m	1–10/d	Gelastic, motor focal	**Interictal**: left, fronto-centro-parietal spike-waves	3	PP	47 × 30 × 27	Tuber,/mamillary body I.V./E.V. Sellar/parasellar	M	Medtronic Stealth Autoguide^TM^	No		0	IV
								**Ictal**: left EEG flattening										
#8	M	HH	0,2 m	42	4 y 3 m	1–5/d	Gelastic, motor focal	**Interictal**: Posterior bilateral SpWs (right>left)	3	ID, PP	20 × 23 × 24	Tuber,/mamillary body I.V/E.V.	R	Medtronic Stealth Autoguide^TM^	Yes	No	0	Ia
								**Ictal**: diffuse EEG flattening										

*HH, Hypothalamic Hamartoma; DNET, dysembryoplastic neuroepithelial tumor; TS Tuberous Sclerosis; Compl., complications; ASM, anti-seizure medications; ID, Intellectual Disability; DD, developemental Delay; PP, Precocious Puberty; ASD, autism spectrum disorder; APOS, acute postoperative seizures; E.V., extra-ventricular; I.V, intra-ventricular CT, centro-temporal; FT, fronto-temporal; T, temporal; M, median; L, left; Cardiac malf., cardiac malformation; SpWs, spike-waves; SlWs, slow waves*.

Overall, the age of the patients ranged from 11 months to 15 years. The mean age at surgery was 6 years (73 months). Six of eight (75%) patients were male. Pathologies were represented by HH in six patients, TSC in one case, and DNET (residual lesion) in another one. Precocious puberty was present in three patients with HH (pt nr. 1, 7, 8); two of them received pharmacological treatment before surgical intervention.

Patients were studied with prolonged video-EEG monitoring and brain MRI.

Preoperative seizure frequencies ranged from 1 event per month to more than 40 per day. Seizure types were gelastic and dacrystic seizure in the six HH patients, five of them presented with additional seizure types (two focal seizures with impaired awareness—patient nr. 1 and 4—and one with spasms—pt. nr. 2). In patient nr 7 and 8, we could not assess the level of awareness impairment during the seizure (young age in pt 7, intellectual disability in pt 8). Patients affected with DNET and TSC presented with focal seizures with preserved awareness.

In the HH cases, the EEG data, together with anatomical localization of the hamartoma, were used to decide the side of the entry point of the laser fiber on the skull.

**Figure 1:**
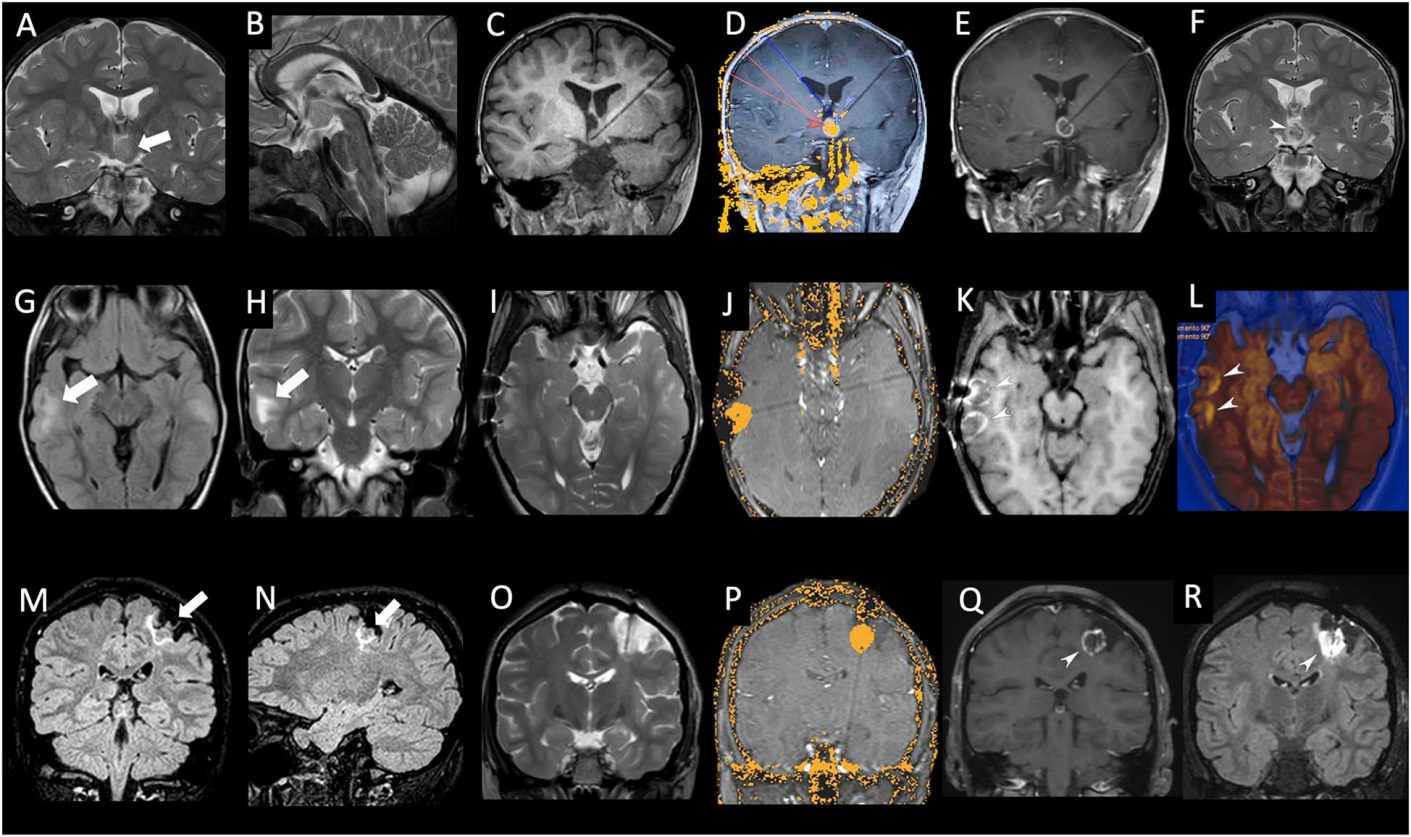
**(A–F):** Patient #2 (see Table 1), male, 2 years old. Coronal **(A)** and sagittal **(B)** T2-weighted images showing the intraventricular hypothalamic hamartoma localized on the left side (thick white arrow). Coronal T1-weighted image **(C)** acquired after stereotactic placement of a laser cannula within the hamartoma. Real-time MR thermogram overlaid on background T1-weighted image **(D)** exhibiting the irreversible damage map (yellow area within the hamartoma). Note the low-limit threshold, set at 48°C (blue arrowhead), placed on the left mammillothalamic tract, and the high limit-thresholds, set at 90°C (red arrowhead) at the tip of laser catheter and within the hamartoma. Coronal post-contrast T1-weighted image **(E)** performed at the end of laser ablation showing central necrosis of ablated hamartoma with peripheral contrast enhancement. Coronal T2-weighted image **(F)** performed 48 h after laser ablation confirming necrosis of ablated hamartoma (white arrowhead). **(G–L):** Patient #5 (see Table 1), male, 6 years old with tuberous sclerosis. Axial FLAIR **(G)** and coronal T2-weighted **(H)** image showing two cortical tubers in the right temporal lobe (white arrow). Axial T2-weighted image **(I)** acquired after stereotactic placement of two laser cannulas within the cortical tubers. Real-time MR thermogram overlaid on background T1-weighted image **(J)** exhibiting the irreversible damage map (yellow area within the tuber). Axial post-contrast T1-weighted image **(K)** performed at the end of laser ablation showing central necrosis of ablated tubers with peripheral contrast enhancement. Axial diffusion weighted image **(L)** overlaid on the axial T2-weighted image, performed at the end of laser ablation confirming necrosis of ablated tubers with peripheral restricted diffusion (white arrowheads). **(M–R):** Patient #6 (see Table 1), female, 15 years old. Coronal **(M)** and sagittal **(N)** FLAIR images showing relapsing DNET localized in the left post-central gyrus (thick white arrows). Coronal T2-weighted image **(O)** acquired after stereotactic placement of a laser cannula within the lesion. Real-time MR thermogram overlaid on the background T1-weighted image **(P)** exhibiting the irreversible damage map (yellow area within the hamartoma). Coronal post-contrast T1-weighted **(Q)** and FLAIR image **(R)** performed at the end of laser ablation showing central necrosis of ablated lesion with peripheral contrast enhancement (white arrowhead).

The trajectories of the laser fiber were designed using the following principles. For the HH cases, the target point of the laser probe was placed at a point between the lateral two-thirds and the mesial third of the maximum diameter of the lesion, measured on coronal and axial planes. Then, the intralesional target point was shifted slightly in a caudal and dorsal direction in the sagittal plane to maximize heat dissipation by the “heat sinks,” represented by the basal arachnoid cisterns and blood vessels. The trajectories of the laser probe were planned in order to avoid structures that could potentially be damaged mechanically or by the heat developed by the laser, such as cerebral blood vessels, optic tracts and/or optic chiasm, mammillothalamic tracts, and fornices. The laser system used (Medtronic Visualase™) made it possible to monitor in almost real time the temperature variations reached at certain points, in order to avoid damage to the perilesional nervous structures, automatically stopping laser delivery in the event of an increase in temperature beyond a previously set level (usually 45°C; see also Figure 1). In the patient with TSC, we performed a double ablation of two cortical tubers, during a single therapeutic session, using two laser fibers, monitoring temperature increments to protect ipsilateral optical radiation. In the case of treatment of residual parietal DNET, we paid special attention to avoid dangerous temperature increases at the level of the corticospinal tract. Postoperative outcomes after laser ablation, as characterized by the Engel Epilepsy Surgery Outcome Scale, ranged from class I to class IV.

Three of the eight patients (two HH, one DNET) were completely seizure-free after the procedure.

One patient (HH) experienced two focal episodes in the 2 months after the procedure; to date, he is seizure-free with only one anti-seizure medication (ASM).

Two patients with HH experienced rare, non-disabling seizures after the procedure (classified as Engel Ib). One patient with HH (#7) showed no seizure improvement (it is worth noting that the patient presented with a large HH).

In the TSC patient with drug-resistant epilepsy associated with multiple cortical tubers, a significant reduction in seizure frequency (from two to three episodes per day to <1 episode per week) was achieved, along with an improvement in his behavioral hyperactivity.

Postoperatively, no patients experienced new neurological morbidity or endocrine dysfunction, with two of them experiencing acute postoperative seizures (APOS). The mean hospital stay was 6 days, and 100% of patients regained normal preoperative motility and activity on the second day after the procedure. In three patients, neurodevelopmental assessment (Vineland scales) before and after the surgical procedure (at 6 months in one patient, at 12 months in 2 patients) showed improvement in cognitive and social behavior.

Although follow-up is overall very short (in two cases is shorter than 3 months) and the number of patients is still quite small, our data are globally consistent with those reported in literature, showing that the high majority of HH patients become seizure free after the procedure (15, 17). Only one patient with a large HH has not shown an improvement after the intervention.

In our opinion, besides from seizure outcome, the most striking aspect of the procedure is the regain of habitual motility and functioning in such a short time compared to traditional surgery, with a reduced hospital stay and convalescence time (mean hospital stay 6 days vs. 12 days).

The relevance of the technique is also fundamental in treatment of multiple tubers as in TSC cases: such a minimally invasive procedure could potentially make it possible to perform multiple surgeries on multiple tubers, something that is not advisable with a classical open technique.

## Discussion/General Considerations/Conclusion

Although the evidence on the therapeutic results of LiTT in the pediatric setting is still limited in terms of both quantity and scientific quality, there is no doubt that, at least in perspective, it may represent a first line of minimally invasive treatment of diseases associated with drug-resistant epilepsy. This consideration is particularly pertinent for those brain lesions difficult to access with the methods of traditional surgery. The overall complication rate is considered more than acceptable compared to traditional surgical techniques, the epileptological outcome obtained with LiTT is commonly evaluated as good. It remains to be clarified what will be the real economic costs of this innovative technique on the various health systems. Carefully designed scientific studies will in any case have to take into account not only the currently high costs of this new technology but also the indirect cost savings through a shorter duration of hospitalizations, cost savings on ASMs, and medium- and long-term follow-up. Ultimately, although future prospective, multicenter studies will better define the role of LiTT in neurosurgery, it is more than reasonable to believe that, with increasing ease of use and a more robust demonstration of efficacy, LiTT will rapidly become an extremely attractive therapeutic method for the treatment of many conditions associated with drug-resistant epilepsy.

## Data Availability Statement

The original contributions presented in the study are included in the article/supplementary material, further inquiries can be directed to the corresponding author.

## Ethics Statement

Ethical review and approval was not required for the study on human participants in accordance with the local legislation and institutional requirements. Written informed consent to participate in this study was provided by the participant's legal guardian/next of kin.

## Author Contributions

AC: ideation, data collection and analysis, writing and critical revision of the paper. EC, MP, and DT: data collection and critical revision of the paper. MM, GD, and GP: critical revision of the paper. LN: data analysis and critical revision of the paper. All authors contributed to the article and approved the submitted version.

## Funding

DINOGMI contributed to this work within the framework of the DINOGMI Department of Excellence MIUR 2018-2022 (legge 232 del 2016).

## Conflict of Interest

The authors declare that the research was conducted in the absence of any commercial or financial relationships that could be construed as a potential conflict of interest.

## Publisher's Note

All claims expressed in this article are solely those of the authors and do not necessarily represent those of their affiliated organizations, or those of the publisher, the editors and the reviewers. Any product that may be evaluated in this article, or claim that may be made by its manufacturer, is not guaranteed or endorsed by the publisher.
